# Crystal structure and magnetic properties of bis­[butyl­tris­(1*H*-pyrazol-1-yl)borato]iron(II)

**DOI:** 10.1107/S2056989019011137

**Published:** 2019-08-20

**Authors:** Maksym Seredyuk, Kateryna Znovjyak, Igor O. Fritsky, Tatiana Y. Sliva, Mykola S. Slobodyanik

**Affiliations:** a Department of Chemistry, Taras Shevchenko National University of Kyiv, Volodymyrska Street 64, Kyiv, 01601, Ukraine; b UkrOrgSyntez Ltd, Chervonotkatska Street 67, Kyiv 02094, Ukraine

**Keywords:** crystal structure, polypyrazolyl borate, iron(II) scorpionates, magnetism, C—H⋯π inter­actions

## Abstract

The asymmetric unit of the title compound, [Fe(C_13_H_18_BN_6_)_2_], contains two half independent complex mol­ecules. In each complex, the Fe^II^ atom is located on an inversion center and is surrounded by two scorpionate ligand butyl­tris­(1*H*-pyrazol-1-yl)borate mol­ecules that coordinate to the iron(II) ion through the N atoms of the pyrazole groups. The two independent complex mol­ecules differ essentially in the conformation of the butyl substituents. In the crystal, the complex mol­ecules are linked by a series of C—H⋯π inter­actions, which generate a supra­molecular three-dimensional structure. At 120 K, the average Fe—N bond distance is 1.969 Å, indicating the low-spin state of the iron(II) atom, which does not change upon heating, as demonstrated by high-temperature magnetic susceptibility measurements.

## Abstract 2

The title compound, bis­[butyl­tris­(1*H*-pyrazol-1-yl)borato]iron(II), belongs to the class of neutral scorpionate complexes. The crystal structure at low temperature and the magnetic susceptibility measurement in the high-temperature region reveal the low-spin character of the iron(II) ion.

## Chemical context   

Scorpionates, coordination metal complexes of poly(1-pyrazol­yl)borates, have been studied intensively since the pioneering work of Trofimenko (1999 ▸). Iron(II) derivatives are particularly inter­esting because of the spin-state crossover between ^1^ *A*_1_ low-spin (LS) and ^5^ *T*_2g_ high-spin (HS) observed for several scorpionate ligands (Long *et al.*, 2004 ▸; Halcrow, 2007 ▸). Complexes of this type are sensitive to the effects induced by substituents on the electronic structure of the ligand and/or steric crowding (Hamon *et al.*, 2008 ▸). The prototypical [Fe(HB(pz)_3_)_2_], the LS compound at 295 K, undergoes a spin-state crossover to the HS state upon heating to *ca* 420 K (Long *et al.*, 2004 ▸). Introducing methyl substituents to the pyrazole moieties decreases the ligand field and shifts the spin crossover down in temperature or completely stabilizes the high-spin state of the iron(II) ion (Long *et al.*, 2004 ▸). In contrast, scorpionate ligands bearing an organic substituent instead of the hydrogen atom on the hub boron atom demonstrate stabilization of the low-spin state and shift of the spin transition to the higher temperature range (Hamon *et al.*, 2008 ▸).

Our continuing inter­est consists of a study of iron(II) complexes bearing alkyl chains (Seredyuk, 2012 ▸; Seredyuk *et al.*, 2006 ▸, 2010 ▸, 2016 ▸) and those based on azol ligands (Seredyuk *et al.*, 2007 ▸, 2015 ▸). Here we report on the synthesis, crystal structure and magnetic properties of an alkyl­ated charge-neutral iron(II) complex based on the scorpionate ligand butyl­tris­(1*H*-pyrazol-1-yl)borate.
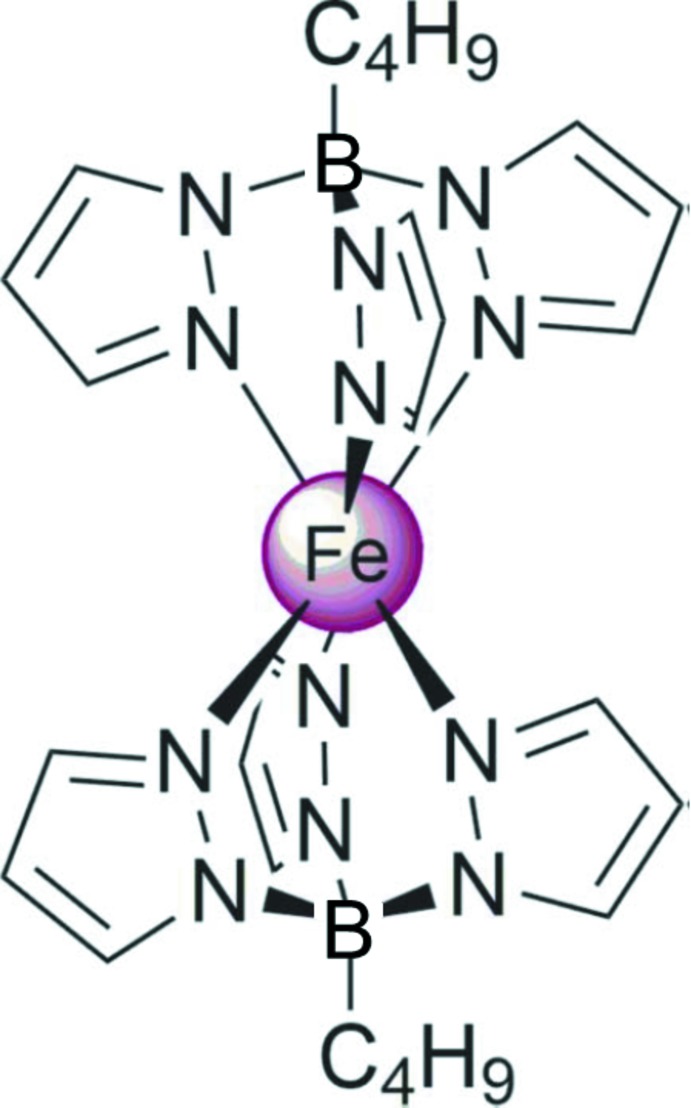


## Structural commentary   

**Figure 1:**
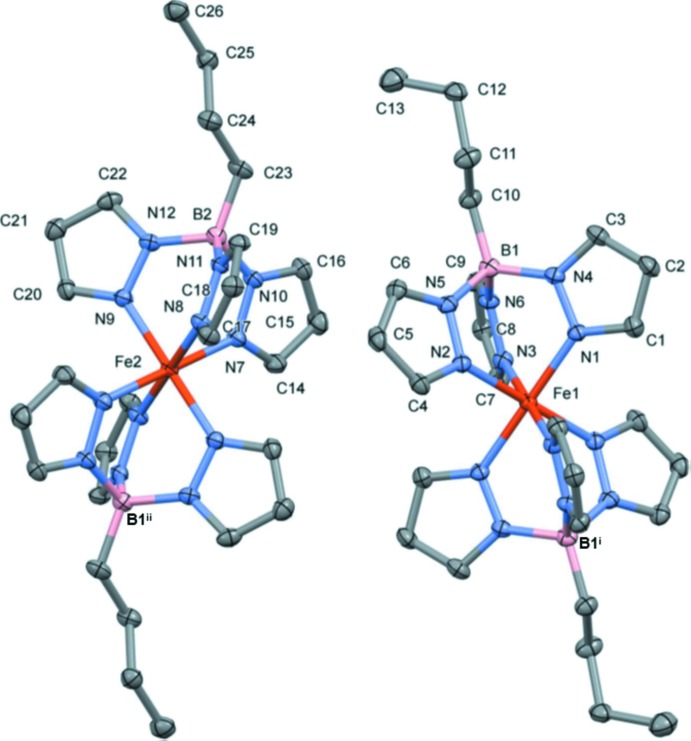
Mol­ecular structure of the two complex mol­ecules of the title compound showing the atom labelling [symmetry codes: (i) −*x*, −*y*, −*z* + 1; (ii) −*x* + 1, −*y*, −*z* + 1]. Displacement ellipsoids are drawn at the 50% probability level. For clarity, H atoms have been omitted.

The asymmetric unit of the title compound contains two half independent complex mol­ecules. In each complex, the Fe^II^ atom, that is located on an inversion center, is surrounded by two scorpionate ligands; each one providing three pyrazole moieties coordinated in a *fac* mode, thus a pseudo-octa­hedral [FeN_6_] coordination polyhedron is formed (Fig. 1 ▸). The two complex mol­ecules differ essentially in the conformation of the butyl groups. One of the methyl­ene groups of the butyl substituents of the Fe1-based complex shows a *gauche* conformation, whilst the remaining two methyl­ene groups are in the *trans* conformation. Oppositely, the three methyl­ene groups of the butyl substituent of the Fe2-based mol­ecule are close to a *trans* conformation (Fig. 1 ▸).

The average Fe—N bond length is 1.969 Å (Table 1 ▸), a typical value for the low-spin state of the iron(II) ion (Gütlich & Goodwin, 2004 ▸). The average trigonal distortion parameters *Φ =* Σ_1_^24^(60 - *θ*_i_)/24, where *θ*_i_ is the angle generated by superposition of two opposite faces of the octa­hedron (Chang *et al.* 1990 ▸,) and *Σ* = Σ_1_^12^(|*ϕ*_i_ − 90|), where *ϕ*_i_ are the deviations from 90° of the *cis*-N—Fe—N angles in the coordination sphere (Drew *et al.* 1995 ▸), are 1.27 and 24.38°, respectively, which correspond to a relatively low distortion of the coordination polyhedron and are typical for the low-spin state of iron(II) (Guionneau *et al.*, 2004 ▸). The averaged volume of the coordination polyhedron is equal to 10.155 Å^3^.

## Supra­molecular features   

**Figure 2:**
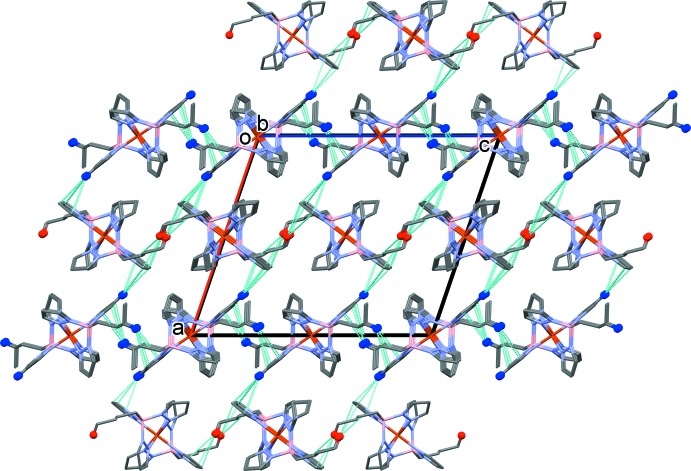
A view along the *b* axis of the crystal packing of the title compound, with the C—H⋯π contacts (see Table 2 ▸ for details) represented by dashed lines. For clarity, only the H atoms involved are shown, as blue balls for the Fe1 complex mol­ecule and red balls for the Fe2 complex mol­ecule.

In the crystal, mol­ecules are linked by C—H⋯π inter­actions (Fig. 2 ▸, Table 2 ▸). The pyrazole–butyl or but­yl–butyl contacts link the individual complex mol­ecules to form layers parallel to the *bc* plane. The layers are linked by C—H⋯π pyrazole–pyrazole inter­actions (Table 2 ▸), leading to the formation of a supra­molecular three-dimensional structure, as shown in Fig. 2 ▸.

## Magnetic measurements   

**Figure 3:**
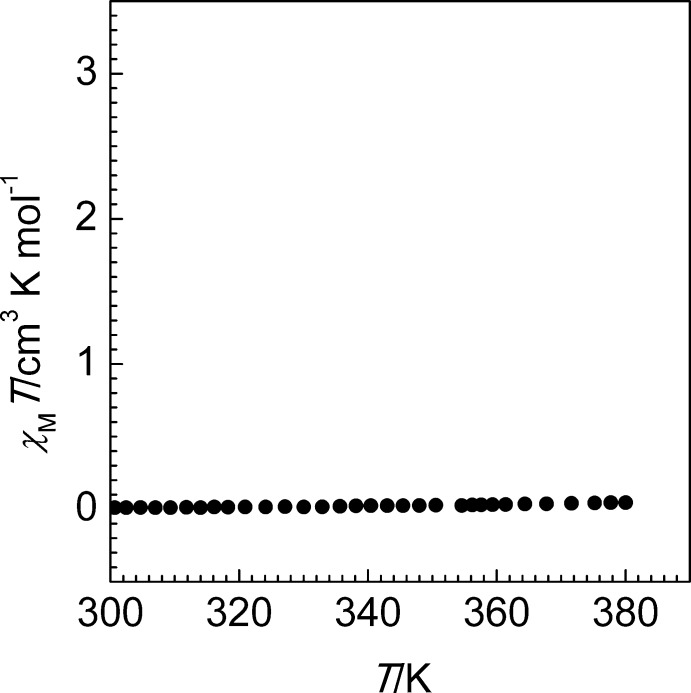
*χ*_M_ *T versus T* plot for the title compound.

Variable-temperature magnetic susceptibility measurements were performed on single crystals (20 mg) of the title compound using a Quantum Design MPMS2 superconducting quantum inter­ference device (SQUID) susceptometer operating at 1 T in the temperature range 10–400 K. Experimental susceptibilities were corrected for the diamagnetism of the holder (gelatine capsule) and of the constituent atoms by the application of Pascal’s constants. The magnetic behaviour of the compound recorded at 2 K min^−1^ is shown in Fig. 3 ▸ in the form of *χ*_M_ *T versus T* (*χ*_M_ is the molar magnetic susceptibility and *T* is the temperature). At 300 K, the *χ*_M_ *T* value is close to zero, and on heating the value remains constant up to 400 K. This corroborates well with the observed short average Fe—N bond length at 120 K and identifies the low-spin state of the central iron(II) ion.

## Database survey   

A search of the Cambridge Structural Database (CSD, Version 5.39, update November 2017; Groom *et al.*, 2016 ▸) for complexes containing the iron(II) ion based on a scorpionate ligand with a tri(1*H*-pyrazol-1-yl)borate fragment yielded 39 hits, with Fe—N bond lengths lying in the ranges 1.956–1.995 and 2.162–2.246 Å, respectively, for the low- and high-spin states of the iron(II) ion.

## Synthesis and crystallization   

The butyl­tris­(1*H*-pyrazol-1-yl)borate ligand and the title compound were synthesized according to the reported procedures (Reger & Tarquini, 1982 ▸; Myers *et al.*, 2008 ▸). The slow diffusion of hexane vapour into a chloro­form solution of the title compound led to the separation of orange well-shaped crystals.

Elemental analysis for C_26_H_36_B_2_FeN_12_ (found): C, 52.56; H, 6.11; N, 28.29%; (calculated): C, 52.22; H, 6.05; N, 28.38%.

## Refinement   

Crystal data, data collection and structure refinement details are summarized in Table 3 ▸. The H-atoms were included in calculated positions and treated as riding atoms: C—H = 0.93–0.97 Å with *U*_iso_(H) = 1.2*U*_eq_(C).

## Supplementary Material

Crystal structure: contains datablock(s) I. DOI: 10.1107/S2056989019011137/ff2161sup1.cif

Structure factors: contains datablock(s) I. DOI: 10.1107/S2056989019011137/ff2161Isup2.hkl

Supporting information file. DOI: 10.1107/S2056989019011137/ff2161Isup3.cdx

CCDC reference: 1946393

Additional supporting information:  crystallographic information; 3D view; checkCIF report

## Figures and Tables

**Table 1:** Selected bond lengths (Å)

Fe1—N1	1.956 (2)	Fe2—N9	1.963 (2)
Fe1—N2	1.969 (3)	Fe2—N7	1.977 (3)
Fe1—N3	1.971 (2)	Fe2—N8	1.977 (2)

**Table 2:** Hydrogen-bond geometry (Å, °) *Cg*2 and *Cg*11 are the centroids of rings N2/N5/C4–C6 and N8/N11/C17–C19, respectively.

*D*—H⋯*A*	*D*—H	H⋯*A*	*D*⋯*A*	*D*—H⋯*A*
C5—H5⋯*Cg*11	0.93	3.00	3.674 (4)	131
C12—H12*A*⋯*Cg*2^i^	0.97	2.87	3.703 (3)	145
C26—H26*C*⋯*Cg*11^ii^	0.96	2.84	3.720 (4)	153

Symmetry codes: (i) 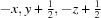; (ii) 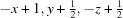.

**Table 3:** Experimental details

Crystal data	
Chemical formula	[Fe(C_13_H_18_BN_6_)_2_]
*M*_r_	594.14
Crystal system, space group	Monoclinic, *P*2_1_/*c*
Temperature (K)	120
*a*, *b*, *c* (Å)	16.0762 (4), 10.1827 (2), 18.3410 (3)
β (°)	108.785 (2)
*V* (Å^3^)	2842.48 (11)
*Z*	4
Radiation type	Mo *K*α
μ (mm^−1^)	0.57
Crystal size (mm)	0.08 × 0.04 × 0.04

Data collection	
Diffractometer	Agilent SuperNova Sapphire3
Absorption correction	Multi-scan (*CrysAlis PRO*; Agilent, 2012 ▸)
*T*_min_, *T*_max_	0.769, 1.000
No. of measured, independent and observed [*I* > 2σ(*I*)] reflections	17726, 7222, 4596
*R*_int_	0.053
(sin θ/λ)_max_ (Å^−1^)	0.700

Refinement	
*R*[*F* ^2^ > 2σ(*F* ^2^)], *wR*(*F* ^2^), *S*	0.053, 0.184, 0.78
No. of reflections	7222
No. of parameters	373
H-atom treatment	H-atom parameters constrained
Δρ_max_, Δρ_min_ (e Å^−3^)	0.39, −0.71

Computer programs: *CrysAlis PRO* (Agilent, 2012 ▸), *SHELXT2014* (Sheldrick, 2015*a* ▸), *Mercury* (Macrae *et al.*, 2008 ▸), *SHELXL2014* (Sheldrick, 2015*b* ▸), *PLATON* (Spek, 2009 ▸) and *publCIF* (Westrip, 2010 ▸).

## supplementary crystallographic information

### Crystal data

**Table:**

[Fe(C_13_H_18_BN_6_)_2_]	*F*(000) = 1248
*M**_r_* = 594.14	*D*_x_ = 1.388 Mg m^−^^3^
Monoclinic, *P*2_1_/*c*	Mo *K*α radiation, λ = 0.71073 Å
*a* = 16.0762 (4) Å	Cell parameters from 2385 reflections
*b* = 10.1827 (2) Å	θ = 3.9–24.7°
*c* = 18.3410 (3) Å	µ = 0.57 mm^−^^1^
β = 108.785 (2)°	*T* = 120 K
*V* = 2842.48 (11) Å^3^	Prismatic, orange
*Z* = 4	0.08 × 0.04 × 0.04 mm

### Data collection

**Table:**

Agilent SuperNova Sapphire3 diffractometer	4596 reflections with *I* > 2σ(*I*)
φ scans and ω scans with κ offset	*R*_int_ = 0.053
Absorption correction: multi-scan (CrysAlis PRO; Agilent, 2012)	θ_max_ = 29.8°, θ_min_ = 2.9°
*T*_min_ = 0.769, *T*_max_ = 1.000	*h* = −21→21
17726 measured reflections	*k* = −12→14
7222 independent reflections	*l* = −23→24

### Refinement

**Table:**

Refinement on *F*^2^	Primary atom site location: dual
Least-squares matrix: full	Secondary atom site location: difference Fourier map
*R*[*F*^2^ > 2σ(*F*^2^)] = 0.053	Hydrogen site location: inferred from neighbouring sites
*wR*(*F*^2^) = 0.184	H-atom parameters constrained
*S* = 0.78	*w* = 1/[σ^2^(*F*_o_^2^) + (0.1086*P*)^2^ + 7.1302*P*] where *P* = (*F*_o_^2^ + 2*F*_c_^2^)/3
7222 reflections	(Δ/σ)_max_ = 0.042
373 parameters	Δρ_max_ = 0.39 e Å^−^^3^
0 restraints	Δρ_min_ = −0.71 e Å^−^^3^

### Special details

**Table:**

Experimental. CrysAlisPro, Agilent Technologies, Version 1.171.36.21 (release 14-08-2012 CrysAlis171 .NET) (compiled Sep 14 2012,17:21:16) Empirical absorption correction using spherical harmonics, implemented in SCALE3 ABSPACK scaling algorithm.
Geometry. Bond distances, angles etc. have been calculated using the rounded fractional coordinates. All su's are estimated from the variances of the (full) variance-covariance matrix. The cell esds are taken into account in the estimation of distances, angles and torsion angles

### Fractional atomic coordinates and isotropic or equivalent isotropic displacement parameters (Å^2^)

**Table:**

	*x*	*y*	*z*	*U*_iso_*/*U*_eq_	
Fe1	0.00000	0.00000	0.50000	0.0155 (2)	
Fe2	0.50000	0.00000	0.50000	0.0164 (2)	
N1	−0.08358 (16)	0.1221 (2)	0.43205 (13)	0.0180 (7)	
N2	0.07144 (17)	0.0187 (2)	0.43129 (14)	0.0190 (7)	
N3	0.06259 (17)	0.1526 (2)	0.55863 (13)	0.0186 (7)	
N4	−0.05505 (17)	0.2339 (2)	0.40633 (13)	0.0191 (7)	
N5	0.09021 (17)	0.1412 (3)	0.40957 (14)	0.0195 (7)	
N6	0.07608 (17)	0.2625 (2)	0.52172 (13)	0.0185 (7)	
C1	−0.1716 (2)	0.1239 (3)	0.40482 (17)	0.0233 (9)	
C2	−0.2013 (2)	0.2375 (3)	0.36109 (18)	0.0256 (9)	
C3	−0.1259 (2)	0.3041 (3)	0.36344 (17)	0.0238 (9)	
C4	0.1166 (2)	−0.0672 (3)	0.40370 (17)	0.0232 (9)	
C5	0.1659 (2)	−0.0017 (3)	0.36471 (18)	0.0257 (10)	
C6	0.1480 (2)	0.1291 (3)	0.37018 (17)	0.0219 (9)	
C7	0.0940 (2)	0.1777 (3)	0.63425 (16)	0.0211 (8)	
C8	0.1281 (2)	0.3052 (3)	0.64677 (17)	0.0212 (8)	
C9	0.1151 (2)	0.3554 (3)	0.57409 (17)	0.0215 (8)	
C10	0.0694 (2)	0.3998 (3)	0.40007 (17)	0.0229 (9)	
C11	0.0458 (2)	0.4211 (3)	0.31252 (17)	0.0281 (10)	
C12	0.0626 (2)	0.5604 (3)	0.28979 (18)	0.0263 (9)	
C13	0.1591 (3)	0.5980 (4)	0.3142 (2)	0.0374 (11)	
B1	0.0458 (2)	0.2628 (4)	0.43210 (18)	0.0196 (9)	
N7	0.42396 (17)	0.1237 (3)	0.53253 (14)	0.0206 (7)	
N8	0.42220 (17)	0.0242 (2)	0.39276 (14)	0.0191 (7)	
N9	0.56735 (17)	0.1520 (2)	0.48428 (14)	0.0199 (7)	
N10	0.39344 (17)	0.2352 (2)	0.49125 (14)	0.0200 (7)	
N11	0.39382 (16)	0.1463 (2)	0.36572 (14)	0.0175 (7)	
N12	0.52540 (17)	0.2641 (2)	0.45014 (14)	0.0195 (7)	
C14	0.3881 (2)	0.1201 (3)	0.58890 (18)	0.0254 (9)	
C15	0.3332 (2)	0.2279 (4)	0.58413 (19)	0.0292 (10)	
C16	0.3382 (2)	0.2988 (3)	0.52117 (17)	0.0229 (9)	
C17	0.3881 (2)	−0.0589 (3)	0.33497 (17)	0.0222 (9)	
C18	0.3368 (2)	0.0083 (3)	0.26958 (18)	0.0234 (9)	
C19	0.3418 (2)	0.1382 (3)	0.29165 (17)	0.0214 (8)	
C20	0.6534 (2)	0.1765 (3)	0.50184 (17)	0.0228 (9)	
C21	0.6682 (2)	0.3032 (3)	0.48037 (18)	0.0262 (9)	
C22	0.5852 (2)	0.3555 (3)	0.44766 (17)	0.0241 (9)	
C23	0.3840 (2)	0.4040 (3)	0.38150 (18)	0.0245 (9)	
C24	0.4056 (2)	0.4516 (3)	0.30977 (17)	0.0240 (9)	
C25	0.4168 (2)	0.6000 (3)	0.30874 (18)	0.0244 (9)	
C26	0.4399 (2)	0.6506 (3)	0.2394 (2)	0.0299 (10)	
B2	0.4229 (2)	0.2666 (3)	0.42016 (19)	0.0194 (9)	
H1	−0.20780	0.05890	0.41370	0.0280*	
H2	−0.25920	0.26240	0.33600	0.0310*	
H3	−0.12370	0.38400	0.33960	0.0280*	
H4	0.11540	−0.15790	0.40960	0.0280*	
H5	0.20280	−0.03860	0.34030	0.0310*	
H6	0.17170	0.19810	0.35010	0.0260*	
H7	0.09340	0.11890	0.67290	0.0250*	
H8	0.15380	0.34700	0.69380	0.0250*	
H9	0.13070	0.43920	0.56300	0.0260*	
H10A	0.04100	0.46890	0.41980	0.0280*	
H10B	0.13220	0.41290	0.42270	0.0280*	
H11A	0.07970	0.36020	0.29270	0.0340*	
H11B	−0.01580	0.40030	0.28820	0.0340*	
H12A	0.03790	0.56890	0.23430	0.0320*	
H12B	0.03210	0.62170	0.31270	0.0320*	
H13A	0.16460	0.68650	0.29830	0.0450*	
H13B	0.18960	0.53960	0.29040	0.0450*	
H13C	0.18400	0.59160	0.36920	0.0450*	
H14	0.39830	0.05470	0.62610	0.0310*	
H15	0.30040	0.24800	0.61620	0.0350*	
H16	0.30870	0.37670	0.50270	0.0270*	
H17	0.39710	−0.14930	0.33770	0.0270*	
H18	0.30590	−0.02690	0.22170	0.0280*	
H19	0.31410	0.20820	0.26080	0.0260*	
H20	0.69760	0.11660	0.52530	0.0270*	
H21	0.72190	0.34360	0.48660	0.0310*	
H22	0.57260	0.43960	0.42730	0.0290*	
H23A	0.32050	0.39940	0.36780	0.0290*	
H23B	0.40370	0.47130	0.42070	0.0290*	
H24A	0.45930	0.40960	0.30860	0.0290*	
H24B	0.35870	0.42520	0.26390	0.0290*	
H25A	0.36270	0.64140	0.30930	0.0290*	
H25B	0.46280	0.62610	0.35530	0.0290*	
H26A	0.44570	0.74440	0.24240	0.0360*	
H26B	0.39420	0.62690	0.19290	0.0360*	
H26C	0.49440	0.61230	0.23920	0.0360*	

### Atomic displacement parameters (Å^2^)

**Table:**

	*U*^11^	*U*^22^	*U*^33^	*U*^12^	*U*^13^	*U*^23^
Fe1	0.0161 (3)	0.0176 (3)	0.0129 (3)	0.0013 (2)	0.0048 (2)	0.0009 (2)
Fe2	0.0173 (3)	0.0149 (3)	0.0166 (3)	0.0033 (2)	0.0051 (2)	0.0013 (2)
N1	0.0194 (12)	0.0201 (12)	0.0148 (11)	0.0013 (10)	0.0061 (9)	0.0020 (10)
N2	0.0184 (12)	0.0223 (13)	0.0163 (11)	0.0013 (10)	0.0057 (9)	0.0019 (10)
N3	0.0209 (12)	0.0192 (12)	0.0156 (11)	0.0009 (10)	0.0059 (9)	0.0000 (10)
N4	0.0213 (13)	0.0182 (12)	0.0168 (11)	0.0016 (10)	0.0049 (10)	0.0020 (10)
N5	0.0219 (13)	0.0216 (13)	0.0152 (11)	−0.0002 (10)	0.0064 (9)	0.0022 (10)
N6	0.0225 (13)	0.0176 (12)	0.0148 (11)	0.0008 (10)	0.0052 (9)	0.0008 (10)
C1	0.0200 (15)	0.0247 (16)	0.0244 (15)	0.0014 (12)	0.0062 (12)	0.0010 (13)
C2	0.0200 (15)	0.0265 (17)	0.0261 (15)	0.0064 (13)	0.0017 (12)	0.0035 (14)
C3	0.0256 (16)	0.0216 (16)	0.0203 (14)	0.0063 (13)	0.0021 (12)	0.0014 (12)
C4	0.0229 (16)	0.0256 (16)	0.0209 (14)	0.0070 (13)	0.0068 (12)	−0.0007 (13)
C5	0.0232 (16)	0.0352 (19)	0.0218 (15)	0.0076 (14)	0.0114 (12)	0.0010 (14)
C6	0.0217 (15)	0.0277 (17)	0.0168 (13)	−0.0007 (13)	0.0068 (11)	0.0007 (12)
C7	0.0214 (15)	0.0262 (16)	0.0153 (13)	0.0045 (12)	0.0052 (11)	0.0005 (12)
C8	0.0205 (15)	0.0241 (16)	0.0181 (13)	0.0010 (12)	0.0050 (11)	−0.0052 (12)
C9	0.0221 (15)	0.0223 (15)	0.0201 (14)	−0.0033 (12)	0.0069 (12)	−0.0012 (12)
C10	0.0250 (16)	0.0249 (16)	0.0184 (14)	−0.0029 (13)	0.0063 (12)	0.0003 (13)
C11	0.0348 (18)	0.0306 (18)	0.0174 (14)	−0.0065 (15)	0.0065 (13)	−0.0004 (13)
C12	0.0299 (17)	0.0262 (17)	0.0230 (15)	0.0048 (14)	0.0088 (13)	0.0075 (14)
C13	0.040 (2)	0.039 (2)	0.0321 (18)	−0.0056 (17)	0.0101 (16)	0.0066 (17)
B1	0.0233 (17)	0.0221 (17)	0.0139 (14)	0.0010 (14)	0.0067 (12)	0.0001 (13)
N7	0.0216 (13)	0.0199 (13)	0.0198 (12)	0.0046 (10)	0.0058 (10)	0.0027 (10)
N8	0.0209 (13)	0.0164 (12)	0.0191 (12)	0.0039 (10)	0.0051 (10)	0.0000 (10)
N9	0.0206 (13)	0.0176 (12)	0.0207 (12)	0.0013 (10)	0.0055 (10)	0.0020 (10)
N10	0.0203 (12)	0.0174 (12)	0.0219 (12)	0.0050 (10)	0.0063 (10)	0.0011 (10)
N11	0.0191 (12)	0.0160 (12)	0.0177 (11)	0.0035 (10)	0.0065 (9)	0.0020 (10)
N12	0.0226 (13)	0.0154 (12)	0.0199 (12)	0.0013 (10)	0.0059 (10)	0.0014 (10)
C14	0.0303 (17)	0.0257 (16)	0.0233 (15)	0.0061 (14)	0.0128 (13)	0.0063 (13)
C15	0.0294 (18)	0.0345 (19)	0.0278 (16)	0.0075 (15)	0.0151 (14)	−0.0001 (15)
C16	0.0212 (15)	0.0241 (16)	0.0229 (14)	0.0057 (12)	0.0065 (12)	−0.0012 (13)
C17	0.0260 (16)	0.0176 (15)	0.0223 (14)	0.0016 (12)	0.0070 (12)	−0.0036 (12)
C18	0.0245 (16)	0.0224 (16)	0.0210 (14)	−0.0017 (12)	0.0042 (12)	−0.0010 (12)
C19	0.0236 (15)	0.0190 (15)	0.0195 (14)	0.0009 (12)	0.0040 (12)	0.0047 (12)
C20	0.0176 (14)	0.0288 (17)	0.0213 (14)	0.0033 (12)	0.0052 (11)	0.0018 (13)
C21	0.0240 (16)	0.0284 (17)	0.0259 (15)	−0.0044 (13)	0.0077 (13)	−0.0010 (14)
C22	0.0307 (17)	0.0195 (15)	0.0208 (14)	−0.0063 (13)	0.0067 (12)	−0.0009 (12)
C23	0.0306 (17)	0.0177 (15)	0.0227 (15)	0.0064 (13)	0.0051 (13)	0.0004 (12)
C24	0.0296 (17)	0.0204 (15)	0.0206 (14)	0.0036 (13)	0.0061 (12)	0.0012 (13)
C25	0.0272 (17)	0.0207 (15)	0.0268 (15)	0.0010 (13)	0.0108 (13)	−0.0020 (13)
C26	0.0372 (19)	0.0220 (16)	0.0335 (17)	0.0024 (14)	0.0155 (15)	−0.0002 (14)
B2	0.0199 (16)	0.0185 (16)	0.0187 (15)	0.0007 (13)	0.0047 (12)	−0.0011 (13)

### Geometric parameters (Å, º)

**Table:**

Fe1—N1	1.956 (2)	N9—N12	1.370 (3)
Fe1—N2	1.969 (3)	C9—H9	0.9300
Fe1—N3	1.971 (2)	N9—C20	1.339 (4)
Fe1—N1^i^	1.956 (2)	C10—H10A	0.9700
Fe1—N2^i^	1.969 (3)	N10—C16	1.350 (4)
Fe1—N3^i^	1.971 (2)	N10—B2	1.557 (4)
Fe2—N9^ii^	1.963 (2)	C10—H10B	0.9700
Fe2—N9	1.963 (2)	N11—C19	1.349 (4)
Fe2—N7	1.977 (3)	C11—H11A	0.9700
Fe2—N8	1.977 (2)	C11—H11B	0.9700
Fe2—N7^ii^	1.977 (3)	N11—B2	1.554 (4)
Fe2—N8^ii^	1.977 (2)	C12—H12A	0.9700
N1—C1	1.341 (4)	C12—H12B	0.9700
N1—N4	1.367 (3)	N12—C22	1.349 (4)
N2—N5	1.372 (4)	N12—B2	1.560 (4)
N2—C4	1.336 (4)	C13—H13A	0.9600
N3—N6	1.361 (3)	C13—H13B	0.9600
N3—C7	1.339 (4)	C13—H13C	0.9600
N4—B1	1.564 (4)	C14—C15	1.394 (5)
N4—C3	1.360 (4)	C15—C16	1.386 (5)
N5—C6	1.354 (4)	C17—C18	1.396 (4)
N5—B1	1.550 (5)	C18—C19	1.378 (4)
N6—C9	1.350 (4)	C20—C21	1.392 (4)
N6—B1	1.557 (4)	C21—C22	1.382 (5)
C1—C2	1.401 (4)	C23—C24	1.543 (4)
C2—C3	1.378 (5)	C23—B2	1.602 (4)
C4—C5	1.396 (5)	C24—C25	1.523 (4)
C5—C6	1.373 (4)	C25—C26	1.525 (5)
C7—C8	1.399 (4)	C14—H14	0.9300
C8—C9	1.379 (4)	C15—H15	0.9300
C10—C11	1.542 (4)	C16—H16	0.9300
C10—B1	1.606 (5)	C17—H17	0.9300
C11—C12	1.527 (4)	C18—H18	0.9300
C12—C13	1.519 (6)	C19—H19	0.9300
C1—H1	0.9300	C20—H20	0.9300
C2—H2	0.9300	C21—H21	0.9300
C3—H3	0.9300	C22—H22	0.9300
C4—H4	0.9300	C23—H23A	0.9700
C5—H5	0.9300	C23—H23B	0.9700
C6—H6	0.9300	C24—H24A	0.9700
N7—N10	1.365 (4)	C24—H24B	0.9700
N7—C14	1.337 (4)	C25—H25A	0.9700
C7—H7	0.9300	C25—H25B	0.9700
C8—H8	0.9300	C26—H26A	0.9600
N8—C17	1.329 (4)	C26—H26B	0.9600
N8—N11	1.362 (3)	C26—H26C	0.9600
			
N1—Fe1—N2	87.32 (10)	N6—C9—H9	126.00
N1—Fe1—N3	88.38 (9)	C8—C9—H9	126.00
N1—Fe1—N1^i^	180.00	N12—N9—C20	105.8 (2)
N1—Fe1—N2^i^	92.68 (10)	C16—N10—B2	131.2 (2)
N1—Fe1—N3^i^	91.62 (9)	C11—C10—H10B	107.00
N2—Fe1—N3	88.42 (10)	B1—C10—H10B	107.00
N1^i^—Fe1—N2	92.68 (10)	H10A—C10—H10B	107.00
N2—Fe1—N2^i^	180.00	B1—C10—H10A	107.00
N2—Fe1—N3^i^	91.58 (10)	N7—N10—C16	109.8 (2)
N1^i^—Fe1—N3	91.62 (9)	N7—N10—B2	118.9 (2)
N2^i^—Fe1—N3	91.58 (10)	C11—C10—H10A	107.00
N3—Fe1—N3^i^	180.00	N8—N11—C19	109.7 (2)
N1^i^—Fe1—N2^i^	87.32 (10)	N8—N11—B2	119.3 (2)
N1^i^—Fe1—N3^i^	88.38 (9)	C19—N11—B2	131.0 (2)
N2^i^—Fe1—N3^i^	88.42 (10)	C12—C11—H11A	109.00
N7—Fe2—N8^ii^	91.93 (10)	C10—C11—H11A	109.00
N7—Fe2—N9^ii^	91.96 (11)	C10—C11—H11B	109.00
N8—Fe2—N9	87.58 (10)	C12—C11—H11B	109.00
N7^ii^—Fe2—N8	91.93 (10)	H11A—C11—H11B	108.00
N8—Fe2—N8^ii^	180.00	N9—N12—C22	109.8 (3)
N8—Fe2—N9^ii^	92.42 (10)	C11—C12—H12A	109.00
N7^ii^—Fe2—N9	91.96 (11)	C11—C12—H12B	109.00
N8^ii^—Fe2—N9	92.42 (10)	C13—C12—H12A	109.00
N9—Fe2—N9^ii^	180.00	C13—C12—H12B	109.00
N7^ii^—Fe2—N8^ii^	88.07 (10)	H12A—C12—H12B	108.00
N7^ii^—Fe2—N9^ii^	88.04 (11)	C22—N12—B2	131.3 (2)
N8^ii^—Fe2—N9^ii^	87.58 (10)	N9—N12—B2	118.9 (2)
N7—Fe2—N8	88.07 (10)	C12—C13—H13B	109.00
N7—Fe2—N9	88.04 (11)	C12—C13—H13C	109.00
N7—Fe2—N7^ii^	180.00	H13A—C13—H13C	110.00
Fe1—N1—N4	120.8 (2)	H13B—C13—H13C	110.00
Fe1—N1—C1	132.2 (2)	H13A—C13—H13B	109.00
N4—N1—C1	106.9 (2)	C12—C13—H13A	109.00
Fe1—N2—N5	120.13 (19)	N7—C14—C15	110.6 (3)
Fe1—N2—C4	132.79 (19)	C14—C15—C16	104.9 (3)
N5—N2—C4	106.7 (3)	N10—C16—C15	108.1 (3)
Fe1—N3—N6	120.79 (17)	N8—C17—C18	110.5 (3)
Fe1—N3—C7	132.2 (2)	C17—C18—C19	104.8 (3)
N6—N3—C7	106.9 (2)	N11—C19—C18	108.3 (3)
N1—N4—C3	109.0 (3)	N9—C20—C21	111.2 (3)
N1—N4—B1	118.9 (2)	C20—C21—C22	104.5 (3)
C3—N4—B1	132.0 (3)	N12—C22—C21	108.6 (3)
N2—N5—B1	119.0 (3)	C24—C23—B2	119.3 (3)
C6—N5—B1	132.0 (3)	C23—C24—C25	112.5 (3)
N2—N5—C6	109.0 (3)	C24—C25—C26	114.0 (3)
C9—N6—B1	131.7 (3)	N10—B2—N11	105.5 (2)
N3—N6—C9	109.5 (2)	N10—B2—N12	105.8 (2)
N3—N6—B1	118.8 (2)	N10—B2—C23	111.7 (2)
N1—C1—C2	110.4 (3)	N11—B2—N12	106.2 (2)
C1—C2—C3	104.7 (3)	N11—B2—C23	114.2 (2)
N4—C3—C2	108.9 (3)	N12—B2—C23	112.8 (2)
N2—C4—C5	110.4 (3)	N7—C14—H14	125.00
C4—C5—C6	105.0 (3)	C15—C14—H14	125.00
N5—C6—C5	108.9 (3)	C14—C15—H15	128.00
N3—C7—C8	110.1 (3)	C16—C15—H15	128.00
C7—C8—C9	104.8 (3)	N10—C16—H16	126.00
N6—C9—C8	108.6 (3)	C15—C16—H16	126.00
C11—C10—B1	119.7 (3)	N8—C17—H17	125.00
C10—C11—C12	114.2 (3)	C18—C17—H17	125.00
C11—C12—C13	114.0 (3)	C17—C18—H18	128.00
N4—B1—N5	106.9 (3)	C19—C18—H18	128.00
N4—B1—N6	105.1 (2)	N11—C19—H19	126.00
N4—B1—C10	113.4 (3)	C18—C19—H19	126.00
N5—B1—N6	105.4 (2)	N9—C20—H20	124.00
N5—B1—C10	114.4 (3)	C21—C20—H20	124.00
N6—B1—C10	111.0 (3)	C20—C21—H21	128.00
N1—C1—H1	125.00	C22—C21—H21	128.00
C2—C1—H1	125.00	N12—C22—H22	126.00
C1—C2—H2	128.00	C21—C22—H22	126.00
C3—C2—H2	128.00	C24—C23—H23A	108.00
N4—C3—H3	125.00	C24—C23—H23B	108.00
C2—C3—H3	126.00	B2—C23—H23A	107.00
N2—C4—H4	125.00	B2—C23—H23B	108.00
C5—C4—H4	125.00	H23A—C23—H23B	107.00
C4—C5—H5	128.00	C23—C24—H24A	109.00
C6—C5—H5	127.00	C23—C24—H24B	109.00
N5—C6—H6	126.00	C25—C24—H24A	109.00
C5—C6—H6	126.00	C25—C24—H24B	109.00
Fe2—N7—N10	120.6 (2)	H24A—C24—H24B	108.00
N3—C7—H7	125.00	C24—C25—H25A	109.00
C8—C7—H7	125.00	C24—C25—H25B	109.00
N10—N7—C14	106.5 (3)	C26—C25—H25A	109.00
Fe2—N7—C14	132.8 (2)	C26—C25—H25B	109.00
C9—C8—H8	128.00	H25A—C25—H25B	108.00
C7—C8—H8	128.00	C25—C26—H26A	110.00
Fe2—N8—N11	120.43 (17)	C25—C26—H26B	109.00
Fe2—N8—C17	132.88 (19)	C25—C26—H26C	109.00
N11—N8—C17	106.7 (2)	H26A—C26—H26B	109.00
Fe2—N9—N12	120.7 (2)	H26A—C26—H26C	109.00
Fe2—N9—C20	133.5 (2)	H26B—C26—H26C	109.00
			
N2—Fe1—N1—N4	−45.0 (2)	N2—N5—B1—N6	60.6 (3)
N2—Fe1—N1—C1	138.6 (3)	C6—N5—B1—N6	−119.3 (3)
N3—Fe1—N1—N4	43.5 (2)	C6—N5—B1—N4	129.3 (3)
N3—Fe1—N1—C1	−132.9 (3)	C6—N5—B1—C10	2.9 (4)
N2^i^—Fe1—N1—N4	135.0 (2)	N2—N5—B1—C10	−177.3 (2)
N2^i^—Fe1—N1—C1	−41.4 (3)	C9—N6—B1—N5	126.1 (4)
N3^i^—Fe1—N1—N4	−136.5 (2)	N3—N6—B1—N5	−54.5 (3)
N3^i^—Fe1—N1—C1	47.1 (3)	N3—N6—B1—N4	58.3 (3)
N1—Fe1—N2—N5	48.8 (2)	C9—N6—B1—N4	−121.2 (4)
N1—Fe1—N2—C4	−138.9 (3)	B1—N6—C9—C8	179.9 (3)
N3—Fe1—N2—N5	−39.7 (2)	N3—N6—B1—C10	−178.8 (3)
N3—Fe1—N2—C4	132.7 (3)	C9—N6—B1—C10	1.8 (5)
N1^i^—Fe1—N2—N5	−131.2 (2)	N3—N6—C9—C8	0.5 (4)
N1^i^—Fe1—N2—C4	41.1 (3)	N1—C1—C2—C3	0.1 (4)
N3^i^—Fe1—N2—N5	140.3 (2)	C1—C2—C3—N4	0.0 (3)
N3^i^—Fe1—N2—C4	−47.3 (3)	N2—C4—C5—C6	0.0 (4)
N1—Fe1—N3—N6	−42.0 (2)	C4—C5—C6—N5	−0.7 (4)
N1—Fe1—N3—C7	133.5 (3)	N3—C7—C8—C9	0.2 (4)
N2—Fe1—N3—N6	45.4 (2)	C7—C8—C9—N6	−0.4 (4)
N2—Fe1—N3—C7	−139.1 (3)	B1—C10—C11—C12	173.6 (3)
N1^i^—Fe1—N3—N6	138.0 (2)	C11—C10—B1—N5	60.5 (4)
N1^i^—Fe1—N3—C7	−46.5 (3)	C11—C10—B1—N6	179.6 (3)
N2^i^—Fe1—N3—N6	−134.6 (2)	C11—C10—B1—N4	−62.5 (4)
N2^i^—Fe1—N3—C7	40.9 (3)	C10—C11—C12—C13	67.1 (4)
N9—Fe2—N8—N11	−44.3 (2)	Fe2—N7—N10—C16	175.6 (2)
N9—Fe2—N8—C17	135.2 (3)	Fe2—N7—N10—B2	−2.7 (3)
N7^ii^—Fe2—N8—N11	−136.2 (2)	C14—N7—N10—C16	−0.9 (3)
N7^ii^—Fe2—N8—C17	43.3 (3)	C14—N7—N10—B2	−179.3 (3)
N9^ii^—Fe2—N8—N11	135.7 (2)	Fe2—N7—C14—C15	−175.1 (2)
N9^ii^—Fe2—N8—C17	−44.8 (3)	N10—N7—C14—C15	0.8 (4)
N7—Fe2—N9—N12	−42.2 (2)	Fe2—N8—N11—C19	−179.9 (2)
N7—Fe2—N9—C20	136.2 (3)	Fe2—N8—N11—B2	−0.3 (4)
N8—Fe2—N9—N12	46.0 (2)	C17—N8—N11—C19	0.5 (4)
N8—Fe2—N9—C20	−135.7 (3)	C17—N8—N11—B2	−179.9 (3)
N7^ii^—Fe2—N9—N12	137.8 (2)	Fe2—N8—C17—C18	−179.7 (2)
N7^ii^—Fe2—N9—C20	−43.8 (3)	N11—N8—C17—C18	−0.2 (4)
N8^ii^—Fe2—N9—N12	−134.1 (2)	Fe2—N9—N12—C22	178.28 (19)
N8^ii^—Fe2—N9—C20	44.3 (3)	Fe2—N9—N12—B2	−3.0 (3)
N9^ii^—Fe2—N7—C14	41.0 (3)	C20—N9—N12—C22	−0.5 (3)
N7—Fe2—N8—N11	43.8 (2)	C20—N9—N12—B2	178.2 (2)
N7—Fe2—N8—C17	−136.7 (3)	Fe2—N9—C20—C21	−178.0 (2)
N9—Fe2—N7—N10	45.5 (2)	N12—N9—C20—C21	0.6 (3)
N9—Fe2—N7—C14	−139.0 (3)	N7—N10—C16—C15	0.7 (4)
N8^ii^—Fe2—N7—N10	137.8 (2)	B2—N10—C16—C15	178.7 (3)
N8^ii^—Fe2—N7—C14	−46.7 (3)	C16—N10—B2—N12	127.7 (3)
N9^ii^—Fe2—N7—N10	−134.5 (2)	N7—N10—B2—C23	−177.5 (3)
N8—Fe2—N7—N10	−42.2 (2)	C16—N10—B2—C23	4.6 (4)
N8—Fe2—N7—C14	133.3 (3)	C16—N10—B2—N11	−120.0 (3)
Fe1—N1—N4—C3	−177.10 (19)	N7—N10—B2—N12	−54.3 (3)
Fe1—N1—N4—B1	0.0 (3)	N7—N10—B2—N11	57.9 (3)
C1—N1—N4—C3	0.1 (3)	N8—N11—B2—N10	−56.3 (3)
C1—N1—N4—B1	177.3 (2)	C19—N11—B2—N10	123.2 (3)
Fe1—N1—C1—C2	176.6 (2)	C19—N11—B2—C23	0.3 (5)
N4—N1—C1—C2	−0.1 (3)	B2—N11—C19—C18	179.8 (3)
N5—N2—C4—C5	0.7 (3)	C19—N11—B2—N12	−124.7 (3)
Fe1—N2—N5—C6	173.0 (2)	N8—N11—B2—C23	−179.3 (3)
Fe1—N2—N5—B1	−6.9 (3)	N8—N11—B2—N12	55.7 (3)
C4—N2—N5—C6	−1.2 (3)	N8—N11—C19—C18	−0.6 (4)
C4—N2—N5—B1	178.9 (3)	C22—N12—B2—N10	−123.8 (3)
Fe1—N2—C4—C5	−172.4 (2)	N9—N12—B2—N11	−53.9 (3)
C7—N3—N6—C9	−0.4 (4)	N9—N12—B2—N10	57.9 (3)
C7—N3—N6—B1	−179.9 (3)	N9—N12—C22—C21	0.3 (3)
Fe1—N3—C7—C8	−175.8 (2)	B2—N12—C22—C21	−178.2 (3)
N6—N3—C7—C8	0.1 (4)	C22—N12—B2—C23	−1.4 (4)
Fe1—N3—N6—C9	176.2 (2)	C22—N12—B2—N11	124.5 (3)
Fe1—N3—N6—B1	−3.4 (4)	N9—N12—B2—C23	−179.7 (2)
N1—N4—C3—C2	−0.1 (3)	N7—C14—C15—C16	−0.4 (4)
C3—N4—B1—N6	119.8 (3)	C14—C15—C16—N10	−0.2 (4)
B1—N4—C3—C2	−176.7 (3)	N8—C17—C18—C19	−0.2 (4)
N1—N4—B1—N6	−56.6 (3)	C17—C18—C19—N11	0.5 (4)
C3—N4—B1—C10	−1.5 (4)	N9—C20—C21—C22	−0.4 (4)
N1—N4—B1—N5	55.1 (3)	C20—C21—C22—N12	0.1 (3)
C3—N4—B1—N5	−128.5 (3)	B2—C23—C24—C25	−144.3 (3)
N1—N4—B1—C10	−177.9 (2)	C24—C23—B2—N12	61.3 (4)
N2—N5—B1—N4	−50.9 (3)	C24—C23—B2—N10	−179.6 (3)
N2—N5—C6—C5	1.2 (3)	C24—C23—B2—N11	−60.1 (4)
B1—N5—C6—C5	−178.9 (3)	C23—C24—C25—C26	178.9 (3)

Symmetry codes: (i) −*x*, −*y*, −*z*+1; (ii) −*x*+1, −*y*, −*z*+1.

### Hydrogen-bond geometry (Å, º)

*Cg*2 and *Cg*11 are the centroids of rings N2/N5/C4–C6 and N8/N11/C17–C19, respectively.

**Table:**

*D*—H···*A*	*D*—H	H···*A*	*D*···*A*	*D*—H···*A*
C5—H5···*Cg*11	0.93	3.00	3.674 (4)	131
C12—H12*A*···*Cg*2^iii^	0.97	2.87	3.703 (3)	145
C26—H26*C*···*Cg*11^iv^	0.96	2.84	3.720 (4)	153

Symmetry codes: (iii) −*x*, *y*+1/2, −*z*+1/2; (iv) −*x*+1, *y*+1/2, −*z*+1/2.
